# A Descriptive Summary of Tumor-Associated MUC1 (TA-MUC1) Expression in Epithelial Malignancies: A Systematic Review of Case Reports and Case Series

**DOI:** 10.7759/cureus.95636

**Published:** 2025-10-28

**Authors:** Ryuichi Ohta, Kasumi Nishikawa, Kaoru Tanaka, Chiaki Sano, Hidetoshi Hayashi

**Affiliations:** 1 Community Care, Unnan City Hospital, Unnan, JPN; 2 Family Medicine, Unnan City Hospital, Unnan, JPN; 3 Department of Medical Oncology, Kindai University Faculty of Medicine, Osaka, JPN; 4 Community Medicine Management, Shimane University Faculty of Medicine, Izumo, JPN; 5 Department of Medical Oncology, Kindai University Faculty of Medicine, Sayama, JPN

**Keywords:** antibody–drug conjugates (adc), biomarker expression, case report review, epithelial malignancies, immunohistochemistry, metastatic cancer, ta-muc1, tumor-associated muc1

## Abstract

Tumor-associated MUC1 (TA-MUC1) is a hypoglycosylated variant of MUC1 that is aberrantly expressed in many epithelial malignancies. Its limited presence in normal tissues and immunogenic properties suggest potential as a target for antibody-drug conjugates and cancer immunotherapy. However, the clinicopathological spectrum and therapeutic context of TA-MUC1-positive tumors remain insufficiently characterized. We conducted a systematic review of case reports and case series describing immunohistochemically confirmed TA-MUC1 expression in malignant tumors. A total of 73 individual cases from 61 publications were included. Data extraction covered demographics, tumor features, TA-MUC1 expression, treatments, outcomes, and associated biomarkers. TA-MUC1 expression was identified across a wide range of tumor types, most frequently in gastrointestinal, breast, and gynecologic cancers, but also in less common malignancies such as sarcomatoid carcinoma and pleural mesothelioma. Expression was frequently retained in metastatic sites. Immunohistochemical staining varied in intensity and methodology, with strong expression often associated with recurrence or aggressive clinical behavior. Multimodal therapy was commonly reported. Across cases, progression-free survival (PFS) was available in 56 of 73 (76.7%) cases. Among these, 47 of 56 (83.9%) had a documented progression event, whereas nine of 56 (16.1%) were censored (progression-free at last follow-up). The median PFS was 12.0 months (IQR 6.0-19.5; range 0.0-118.0 months). Overall survival (OS) was available in 56 of 73 (76.7%) cases. Among these, 14 of 56 (25.0%) deaths were reported, and 42 of 56 (75.0%) observations were censored (alive at last follow-up). The median OS was 12.0 months (IQR 8.0-26.0; range 0.5-118.0 months). Co-expression with biomarkers such as programmed death-ligand 1 (PD-L1) and human epidermal growth factor receptor 2 (HER2) was occasionally observed. These findings demonstrate that TA-MUC1 has been documented in diverse malignancies, including rare and metastatic presentations. The descriptive evidence supports its potential relevance as a therapeutic target and highlights the need for standardized evaluation and prospective studies.

## Introduction and background

Mucin-1 (MUC1) is a high molecular weight, transmembrane glycoprotein expressed on the apical surface of epithelial cells in various human tissues, where it plays essential roles in cell surface protection, signal transduction, and maintenance of epithelial polarity [1]. In normal tissues, MUC1 is heavily glycosylated and maintains a regular structure; however, in malignant transformation, its glycosylation pattern and expression are substantially altered [2].

Tumor-associated MUC1 (TA-MUC1) refers to an aberrant form of MUC1 specifically expressed in cancer cells. It is characterized by hypoglycosylation, abnormal glycan composition, exposure of the protein core, and disrupted membrane localization [3]. These changes confer distinct antigenic properties compared to normal MUC1, making TA-MUC1 a promising target for cancer immunotherapy and antibody-drug conjugates (ADCs) [4].

TA-MUC1 is widely expressed across multiple epithelial malignancies, including breast, ovarian, pancreatic, gastric, colorectal, and lung cancers. Its expression level has been reported to correlate with higher tumor grade, advanced stage, therapeutic resistance, and poor prognosis [5,6]. Importantly, these findings are largely based on studies employing TA-MUC1-specific immunohistochemistry (IHC) clones (e.g., SM3, Ma695) that recognize hypoglycosylated core peptide epitopes of MUC1 rather than fully glycosylated forms detected by pan-MUC1 antibodies (e.g., HMFG1, HMFG2, KL-6). Additionally, TA-MUC1 contributes to immune evasion within the tumor microenvironment (TME) by suppressing anti-tumor immune responses and potentially reducing the efficacy of immune checkpoint inhibitors [7]. A summary of representative IHC clones and their characteristics is shown in Table 1 to distinguish TA-MUC1-specific antibodies from pan-MUC1 reagents.

**Table 1:** Representative MUC1/TA-MUC1 IHC Clones and Their Characteristics. Abbreviations: VNTR = variable number tandem repeat; IHC = immunohistochemistry; TA = tumor-associated

Clone	Target Epitope / Specificity	Type	Antigen Retrieval	Scoring / Expression Note	
SM3	Exposed PDTRP sequence within the tandem repeat region (hypoglycosylated)	TA-MUC1-specific	Citrate buffer (pH 6.0), microwave	Strongly associated with tumor progression and poor prognosis	
Ma695	Core peptide epitope within VNTR (less glycosylated form)	TA-MUC1-specific	EDTA buffer (pH 8.0), pressure cooker	Detects tumor-restricted MUC1 forms	
HMFG1	Glycosylated VNTR region (general epithelial MUC1)	Pan-MUC1	Citrate buffer (pH 6.0)	Expressed in both normal and malignant epithelial cells	
HMFG2	Overlapping VNTR region (heavily glycosylated)	Pan-MUC1	Citrate buffer (pH 6.0)	Used for total MUC1 expression assessment	
KL-6	Sialylated MUC1 glycoform	Pan-MUC1	Citrate buffer (pH 6.0)	Widely used as serum biomarker; not TA-specific	

Based on these findings, TA-MUC1-targeting ADCs, such as DS-3939a and gatipotuzumab, are currently under investigation in early-phase clinical trials globally. These agents exploit the selective expression of TA-MUC1 on malignant cells to deliver cytotoxic payloads while minimizing off-target toxicity to normal tissues [8].

Despite this therapeutic potential, large-scale data regarding TA-MUC1-positive cancers remain limited. In particular, the distribution of TA-MUC1 across tumor types, its differential expression in primary versus metastatic lesions, and its association with treatment history or response are not well-characterized. Numerous case reports and case series contain valuable insights into TA-MUC1 expression patterns and clinical behavior, but these have yet to be systematically reviewed.

To address this knowledge gap, we conducted a systematic review of published case reports and case series involving TA-MUC1-positive malignancies. Our aim was to comprehensively evaluate tumor-type-specific expression patterns, differences between primary and metastatic lesions, associations with treatment history and outcomes, and rare site involvement. These findings may offer foundational insights for future development of TA-MUC1-targeted therapies and biomarker-based treatment stratification.

## Review

Methods

Study Design

This study was conducted as a systematic review of case reports and case series according to the Preferred Reporting Items for Systematic Reviews and Meta-Analyses (PRISMA) 2020 guidelines for reporting systematic reviews and was prospectively registered with the International Prospective Register of Systematic Reviews (PROSPERO; registration ID: CRD420251114061) [9].

Data Sources and Search Strategy

**Figure 1:**
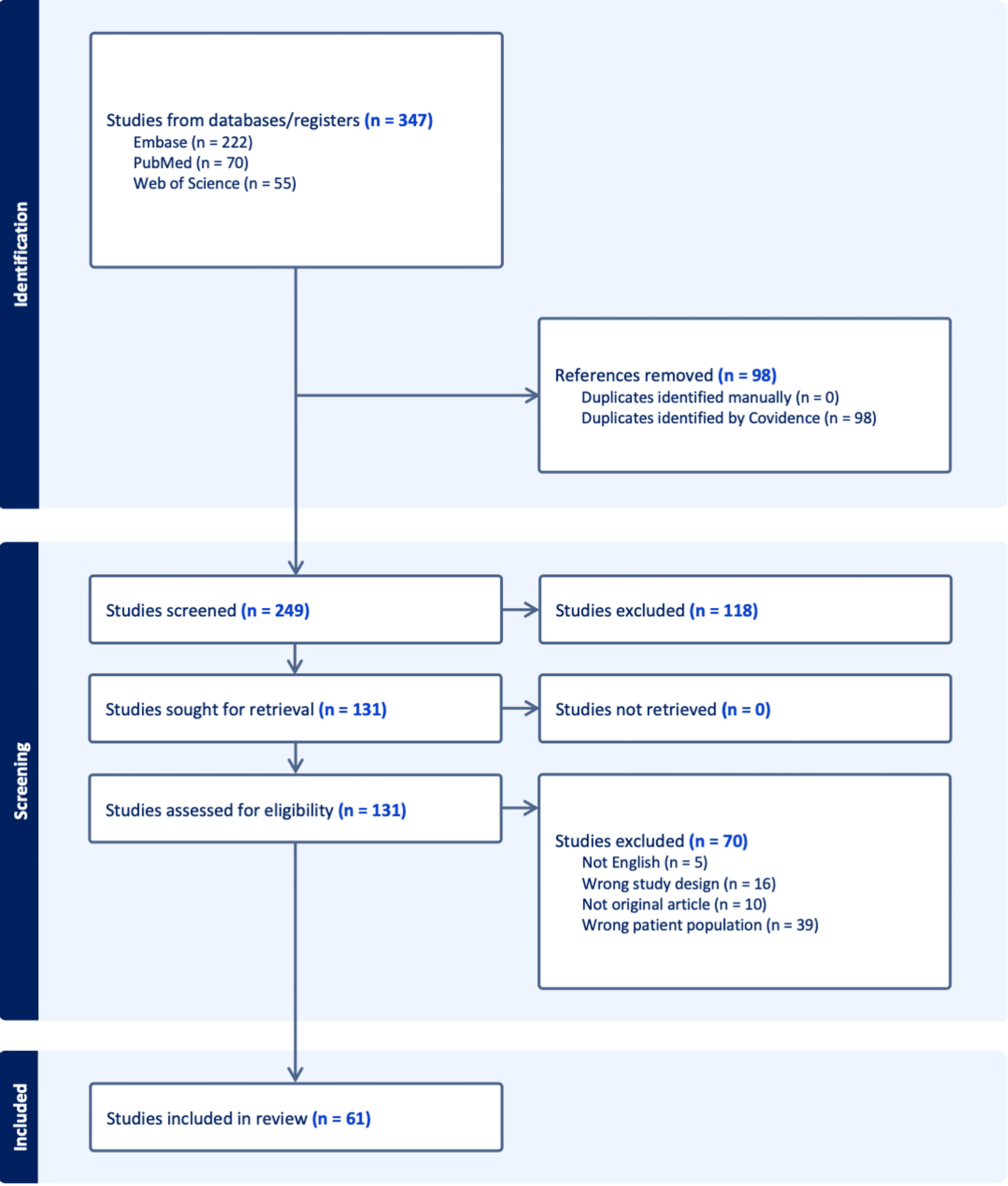
Preferred Reporting Items for Systematic Reviews and Meta-Analyses (PRISMA) Selection Flow

A comprehensive literature search was performed using PubMed, Embase, and Web of Science to identify relevant reports published between January 1, 2000, and June 30, 2025. The following search terms were used: (“TA-MUC1” OR “Tumor-associated MUC1” OR “MUC1” OR “Membrane-associated MUC1” OR “MA-MUC”) AND (“case report” OR “case series”) AND (“cancer” OR “carcinoma” OR “adenocarcinoma” OR “neoplasm”). Searches were limited to studies published in the English language. Grey literature, including conference abstracts and unpublished data, was excluded. The full search strategy and screening flow are illustrated in the PRISMA diagram (Figure 1).

Eligibility Criteria

We applied well-defined inclusion and exclusion criteria to ensure that only clinically informative and histologically verified TA-MUC1-positive malignancies were captured.

Inclusion criteria: We included articles that met all of the following conditions: full-text human case reports or case series that provided patient-level data, diagnosis of a malignant neoplasm, regardless of tumor origin or histological subtype, assessment of TA-MUC1 expression by IHC, with preference for those specifying the antibody used and staining/scoring method, TA-MUC1 expression must have been reported in either primary tumors or metastatic lesions, or both, and detailed clinical information, including treatment course, follow-up, or therapeutic outcomes, had to be available.

Exclusion criteria: We excluded the following types of publications: studies evaluating TA-MUC1 expression only at the transcriptomic or genomic level, without protein-level confirmation by IHC, preclinical studies, including those based solely on cell lines or animal models, abstract-only publications lacking peer-reviewed, full-text detail, articles that did not clearly describe TA-MUC1 expression status or location, duplicate reports from the same patient cohort or institution; in such cases, only the most comprehensive or recent report was retained to avoid data redundancy. These criteria were designed to maximize clinical relevance and ensure that extracted data reflected real-world, tissue-validated TA-MUC1 expression patterns in human cancers.

Study Selection

Two independent reviewers screened titles and abstracts for relevance, followed by a full-text review of potentially eligible articles. A third reviewer resolved discrepancies. The final selection was documented using a PRISMA flowchart.

Data Extraction

Two independent reviewers systematically extracted data from each included publication using a pre-designed, structured Excel template to ensure consistency and reproducibility. For every case, we collected detailed information across multiple domains: publication characteristics, including the first author, year of publication, country of origin, and type of report (e.g., case report or case series), patient-level demographics, such as age, sex, smoking history, and relevant comorbid conditions, tumor characteristics, including the primary cancer type, clinical or pathological stage, and anatomical sites of both primary and metastatic lesions, TA-MUC1-specific information, such as the site of expression (e.g., primary tumor, metastasis, or unusual sites), detection method (immunohistochemical antibody used, staining conditions), level of expression (quantitative or semi-quantitative), and any reported differences between primary and metastatic lesions, treatment history, including details of surgical intervention, systemic chemotherapy, immunotherapy, radiation, hormone therapy, or any TA-MUC1-directed therapeutic strategies, clinical outcomes, including documented tumor response (partial response, stable disease, progressive disease), response duration, follow-up time, survival status, and reported progression-free or overall survival in months, co-existing biomarkers, when available, such as programmed death-ligand 1 (PD-L1), human epidermal growth factor receptor 2 (HER2), microsatellite instability (MSI), and tumor mutational burden (TMB), and additional observations, including any author commentary on unusual TA-MUC1 staining patterns, dynamic changes in expression over time, or detection in rare metastatic sites. This comprehensive extraction allowed for an in-depth descriptive synthesis of clinical behavior, biomarker expression, and outcomes among TA-MUC1-positive malignancies.

Data Synthesis

Given the descriptive nature of the data and expected heterogeneity, a narrative synthesis was conducted. Frequencies and distributions were summarized using descriptive statistics: medians with interquartile ranges (IQR) for continuous variables and percentages for categorical variables. Subgroup trends based on tumor type, expression site, or treatment were explored narratively. No formal meta-analysis or risk-of-bias assessment was performed due to the case-report nature of the data.

Quality Appraisal

Methodological quality was evaluated independently by two reviewers using the Joanna Briggs Institute (JBI) critical appraisal checklist appropriate to each study design (case report: 8 items; case series: 10 items). Each criterion was scored as “Yes,” “No,” “Unclear,” or “Not applicable.” Discrepancies were resolved by discussion with a third reviewer. Inter-rater agreement for initial independent scoring was calculated using Cohen’s κ, yielding κ = 0.84 (95% CI 0.76-0.92), indicating “almost perfect” agreement. For sensitivity assessment, studies rated as “low quality” (≤ 50% items satisfied) were excluded, and descriptive trends were re-examined to evaluate the robustness of key narrative findings.

Results

Study Selection

A total of 347 records were identified through database searches: 222 from Embase, 70 from PubMed, and 55 from Web of Science. After removing 98 duplicate records using Covidence, 249 studies were screened, and 118 studies were excluded based on their titles and abstracts. Through full-text assessment of 131 studies, 70 studies were excluded for the following reasons: not written in English (n = 5), incorrect study design, such as reviews or in vitro studies (n = 16), not original articles (n = 10), or involving the wrong patient population (n = 39). A total of 61 case reports or case series met the inclusion criteria and were included in the final synthesis (Table 2).

**Table 2:** Contents of the Included Articles This table summarizes key clinical and pathological information from individual case reports and case series included in this systematic review. Each entry includes the publication year, first author, country of origin, patient demographics, diagnosis, and clinical course including treatment, progression, recurrence, or survival outcomes. Tumor types include rare subtypes and anatomically diverse primary sites, many of which demonstrated tumor-associated MUC1 (TA-MUC1) expression as reported by immunohistochemical methods in the original articles. This compilation highlights the heterogeneity of TA-MUC1–expressing tumors and their varied clinical trajectories. Abbreviations: IPMT = intraductal papillary mucinous tumor; IDC = invasive ductal carcinoma; ICC = intrahepatic cholangiocarcinoma; MEC = mucoepidermoid carcinoma; IMPC = invasive micropapillary carcinoma; LCIS = lobular carcinoma in situ; SVC = superior vena cava; AFP = alpha-fetoprotein; VATS = video-assisted thoracic surgery; IPMN = intraductal papillary mucinous neoplasms; IVC = inferior vena cava; RFA = Radiofrequency ablation; UC = urothelial carcinoma; IPNB = intraductal papillary neoplasm of the bile duct; SDC = salivary duct carcinoma; ACC = acinar cell carcinoma; DAC = ductal adenocarcinoma; UFT = uracil and tegafur; TUR-BT = transurethral resection of bladder tumor; NET = neuroendocrine tumor; HAC = hepatoid adenocarcinoma; DIC = disseminated intravascular coagulation; RT = radiotherapy; FOLFOX/FOLFIRI = chemotherapy regimens.

First Author, Published Year	Country	Patient Age	Patient Sex	Diagnosis	Clinical Course / Symptom Progression
Higashi M et al., 1999 [10]	Japan	74	Male	Sarcomatoid carcinoma (anaplastic ductal carcinoma) of the pancreas	Presented with epigastric pain → imaging revealed a pancreatic head mass → underwent pylorus-preserving pancreatoduodenectomy → histopathology showed sarcomatoid carcinoma → patient died 3 months post-op from peritoneal carcinomatosis
Jass JR et al., 2000 [11]	Australia	56	Male	Ascending colon adenocarcinoma differentiating as dome epithelium of gut-associated lymphoid tissue (GALT)	Right hemicolectomy performed; no mention of recurrence
Moriya T et al., 2002 [12]	Japan	66	Male	Minute invasive ductal carcinoma (IDC) of the residual pancreas, de novo after prior IPMT	First surgery: distal pancreatectomy for IPMT (carcinoma in situ) 3 years later: recurrent cystic lesion (IPMT adenoma) → duodenum-preserving resection Minute IDC (2 mm) accidentally found 3 months later: hepatoduodenal ligament mass (adenocarcinoma) → intraoperative radiotherapy → patient died of tumor progression
Yamamoto M et al., 2002 [13]	Japan	50	Male	Intrahepatic cholangiocarcinoma (ICC) with lymph node metastasis	Partial hepatectomy + pancreatoduodenectomy + lymph node dissection → no adjuvant chemo → 8 years alive without recurrence
Terada T et al., 2003 [14]	Japan	88	Male	Hepatobiliary cystadenocarcinoma with cystadenoma elements of the gallbladder	Presented with jaundice and hemobilia → imaging revealed multilocular cystic tumor → underwent cholecystectomy → pathology showed cystadenoma with dysplasia and invasive carcinoma cells
Lee WA et al., 2005 [15]	Republic of Korea	67	Male	Gastric extremely well differentiated adenocarcinoma (EWDA) of gastric phenotype	Initially misdiagnosed as benign foveolar hyperplasia on biopsy → radical total gastrectomy → adjuvant chemotherapy → peritoneal carcinomatosis and lung metastases at 12 months → survived 18 months with distant metastases
Sakamoto K et al., 2005 [16]	Japan	67	Male	Sigmoid colon invasive micropapillary carcinoma	Died 18 months after surgery due to peritoneal carcinomatosis
Sakamoto K et al., 2005 [16]	Japan	68	Female	Cecal invasive micropapillary carcinoma	Alive with peritoneal dissemination 1 month after surgery
Sakamoto K et al., 2005 [16]	Japan	53	Female	Ascending colon IMPC	Alive without disease 1 month after surgery
Fadare O et al., 2006 [17]	USA	44	Female	Pleomorphic lobular carcinoma in situ (LCIS) composed almost entirely of signet ring cells	Core biopsy revealed LCIS composed of ~95% signet ring cells with comedo-type necrosis → larger excision performed → no residual disease → no evidence of progression after 26 months
Kusafuka K et al., 2006 [18]	Japan	57	Female	Low-grade mucoepidermoid carcinoma (MEC)	No recurrence/metastasis at 3 months post-op
Kusafuka K et al., 2006 [18]	Japan	51	Female	Low-grade clear cell variant of MEC	No recurrence/metastasis at ~2 months post-op
Yamagata K et al., 2006 [19]	Japan	86	Female	Basal cell adenocarcinoma of minor salivary gland (soft palate)	Radiation 70 Gy → complete disappearance → local recurrence 1 year later → alive with local recurrence at 2 years 2 months
Munakata S et al., 2007 [20]	Japan	73	Female	Micropapillary urothelial carcinoma (MPUC) of the renal pelvis with co-existing conventional papillary urothelial carcinoma (UC)	Detected as a large renal pelvis tumor → underwent left nephrectomy, adrenalectomy, lymph node dissection, and bilateral oophorectomy → postoperative chemotherapy with methotrexate, cisplatin, doxorubicin, and vinblastine → metastases to bone and lymph nodes within 11 months → died of disease 14 months after surgery (no autopsy)
Terada T et al., 2008 [21]	Japan	77	Male	Minute adenocarcinoma arising from Rokitansky–Aschoff sinus	Incidental finding during cholecystectomy, no recurrence after 4 years follow-up
Caruso RA et al., 2008 [22]	Italy	46	Female	Invasive micropapillary carcinoma of the breast	Stable post-mastectomy with tamoxifen, no recurrence reported
Izumi S et al., 2009 [23]	Japan	75	Female	Multiple invasive ductal carcinomas of the pancreas	Four synchronous tumors detected, subtotal pancreatoduodenectomy performed, S-1 given postoperatively; hepatic metastasis at 6 months
Munakata R et al., 2009 [24]	Japan	46	Female	Gingival metastasis of ovarian mucinous cystadenocarcinoma	Gingival lesion appeared 5 years after primary surgery; patient underwent palliative excision of gingival mass; died 18 days later due to acute renal failure
Sonoo H et al., 2009 [25]	Japan	64	Male	Early sigmoid colon cancer with micropapillary carcinoma component	Laparoscopy-assisted sigmoidectomy + lymph node dissection → adjuvant chemotherapy for 25 months → no recurrence
Yaman B et al., 2009 [26]	Turkey	54	Male	Borderline b-IPMN (oncocytic type)	No stromal invasion, borderline lesion
Yaman B et al., 2009 [26]	Turkey	65	Male	Carcinoma in situ b-IPMN (pancreatobiliary type)	Carcinoma in situ, no invasion
Yaman B et al., 2009 [26]	Turkey	60	Male	Invasive carcinoma b-IPMN (pancreatobiliary type)	Invasive tumor with 4 satellite lesions
Mogi A et al., 2009 [27]	Japan	54	Male	Epithelioid malignant mesothelioma with invasive micropapillary component	Underwent VATS biopsy & extrapleural pneumonectomy, developed lymphatic & pulmonary micrometastases
Mogi A et al., 2009 [27]	Japan	73	Female	Epithelioid malignant mesothelioma with invasive micropapillary component	Underwent VATS biopsy & extrapleural pneumonectomy, developed lymphatic & pulmonary micrometastases
Kusafuka K et al., 2010 [28]	Japan	38	Female	Low-grade salivary duct carcinoma (LG-SDC)	Swelling for 2 years, superficial lobectomy performed; no recurrence or metastasis during 8-month follow-up
Yu J et al., 2010 [29]	USA	65	Female	Invasive lobular carcinoma (ILC) of the breast with abundant extracellular mucin production	Initially diagnosed as ductal carcinoma → further workup showed lobular phenotype (loss of E-cadherin, cytoplasmic p120). Surgical resection with sentinel lymph node biopsy performed. Tumor showed discohesive pattern with extracellular mucin.
Ohe M et al., 2011 [30]	Japan	49	Male	Stromal micropapillary pattern predominant lung adenocarcinoma (SMPPLA)	Right upper lobectomy + lymph node dissection → adjuvant chemotherapy → alive without recurrence at 10 months
Mimatsu K et al., 2011 [31]	Japan	60	Male	Mass-forming intrahepatic cholangiocarcinoma with colon invasion and brain metastasis	Tumor detected by US/CT; right hepatectomy + right hemicolectomy → 10 months later brain metastasis → brain metastasis resection + radiotherapy (50 Gy) → oral UFT for 2 years → no recurrence for 7 years
Doi H et al., 2011 [32]	Japan	56	Female	Primary micropapillary carcinoma (MPC) of the transverse colon	Left hemicolectomy with lymph node dissection → adjuvant chemo → multiple para-aortic lymph node metastases after 6 months → treated with FOLFOX + bevacizumab → alive at 15 months
Terada T et al., 2012 [33]	Japan	78	Male	Primary signet-ring cell adenocarcinoma (SRCA) of the lung	Rapid progression → pleural effusion, SVC syndrome → carcinomatosis → death within 3 months
Zen Y et al., 2012 [34]	UK	64	Male	Multiple hilar cysts in explanted liver	No recurrence/metastasis at 5 years
Terada T et al., 2012 [35]	Japan	73	Female	Adenoid squamous cell carcinoma (AdSCC) of the oral cavity	Tumor excision with mandibular bone resection → no recurrence at follow-up
Terada T et al., 2012 [36]	Japan	37	Male	Primary pure signet-ring cell adenocarcinoma (SRCA) of urinary bladder	Died of disease 12 months after diagnosis
Terada T et al., 2012 [36]	Japan	43	Male	Primary pure signet-ring cell adenocarcinoma (SRCA) of urinary bladder	Died of disease 16 months after diagnosis
Terada T et al., 2012 [36]	Japan	84	Female	Primary pure signet-ring cell adenocarcinoma (SRCA) of urinary bladder	Alive 6 months after diagnosis
Kusafuka K et al., 2012 [37]	Japan	84	Female	Mucin-rich salivary duct carcinoma (mSDC) with signet-ring cell feature arising ex pleomorphic adenoma	Tumor excised by submandibulectomy + neck dissection; 8 months later developed bone metastases → radiation for pain control → death 2 months later due to pneumonia/sepsis
Terada T et al., 2013 [38]	Japan	51	Male	Combined hepatocellular-cholangiocarcinoma (C-HCC-CC) with stem cell features, ductal plate malformation (DPM) subtype	Tumor resection → no recurrence reported (follow-up details unclear)
Terada T et al., 2013 [39]	Japan	66	Male	High-grade urothelial carcinoma with multiple divergent differentiations	Diagnosed by TUR-BT → high-grade aggressive tumor with muscular invasion and multiple histologic types → referred to a specialized center
Yamashita S et al., 2013 [40]	Japan	61	Male	Biliary cystadenocarcinoma	Progressive bile duct dilation and solid tumor component → resected
Palermo MH et al., 2013 [41]	Brazil	80	Female	Primary mucoepidermoid carcinoma of the breast	Tumor excised; histology showed high-grade features with frequent mitosis and necrosis, but no vascular/perineural invasion
Luna A et al., 2013 [42]	Argentina	48	Female	Cutaneous metastases from invasive ductal carcinoma of the breast	2010: Primary tumor (ER−/PR−/HER2+) → 2011: Local recurrence treated with mastectomy + chemo → 2012: Multiple skin nodules, systemic metastases detected
Honda Y et al., 2013 [43]	Japan	63	Male	Hidradenocarcinoma with mucinous & squamous differentiation and pagetoid cells	25-year stable nodule → rapid growth → nodal metastasis
Modi Y et al., 2014 [44]	USA	74	Female	Primary clear cell ductal adenocarcinoma of the pancreas with hepatic metastasis	Presented with liver enzyme elevation, large pancreatic mass (6 × 3.5 cm) with multiple hepatic metastases; patient declined chemotherapy and opted for palliative care
You Y et al., 2014 [45]	USA	70	Male	Intestinal-type adenocarcinoma of the gallbladder with precursor tubulopapillary adenoma (ICPN)	Cholecystectomy with lymphadenectomy performed → no lymph node metastasis; precursor adenoma progressed through low- to high-grade dysplasia to invasive carcinoma
Kobayashi M et al., 2014 [46]	Japan	80	Male	Locally recurrent gastric signet-ring adenocarcinoma	Recurrence after multiple resections → TS-1 chemo discontinued → DC vaccine (WT1 & MUC1) → complete regression → remission for 30 months
Behzatoglu K et al., 2014 [47]	Turkey	71	Female	Mucinous urothelial carcinoma of the renal pelvis	Stable, no recurrence/metastasis at 16 months
Furuhata A et al., 2014 [48]	Japan	74	Male	Intraductal tubulopapillary neoplasm (ITPN) of the pancreas with expansile invasive carcinoma	Detected incidentally → EUS-FNA cytology suggested adenocarcinoma → immunoprofile supported ITPN → Whipple resection performed → pathology confirmed ITPN with expansile invasion into duodenal mucosa; no nodal involvement or metastasis reported
Terada T et al., 2015 [49]	Japan	77	Male	Primary minute mucinous adenocarcinoma of appendix (arising from diverticulosis)	Diagnosed as acute appendicitis → appendectomy → no adjuvant chemotherapy → 2.5 years follow-up without metastasis or pseudomyxoma peritonei
Terada T et al., 2016 [50]	Japan	72	Female	Nested variant of urothelial carcinoma (NVUC) of urinary bladder	TUR-BT performed; patient considered cured; on follow-up
Lei L et al., 2016 [51]	USA	53	Female	Serous cystadenocarcinoma of presumed pancreatobiliary differentiation, arising in presumed vitelline duct remnant	Initial mass → extensive workup failed to reveal a primary → treated with XELOX ×3 cycles and gemcitabine/cisplatin ×3 cycles (progression) → surgical resection of abdominal wall mass and lymph nodes (CA19-9 normalized temporarily) → mediastinal lymph nodes treated with radiation → brain metastases appeared 4 weeks post-surgery → patient died 12 months after initial presentation
Fujimoto Y et al., 2017 [52]	Japan	74	Male	Intraductal tubulopapillary neoplasm (ITPN) of the pancreas with associated invasive carcinoma	CT and MRI showed mass with small cystic lesions → endoscopic biopsy revealed adenocarcinoma → total pancreatectomy with splenectomy performed → pathology confirmed ITPN with associated invasive carcinoma → no recurrence at 9-month follow-up
Umemura A et al., 2017 [53]	Japan	50	Female	Intraductal tubulopapillary neoplasm (ITPN) of the pancreatic body, noninvasive adenocarcinoma	Initial presentation with rupture of pancreatic cysts → acute peritonitis due to elevated intraductal pressure → conservative management (drainage + antibiotics) → follow-up imaging revealed 2 cm pancreatic body mass → distal pancreatectomy + radical lymphadenectomy performed → pathology confirmed ITPN with noninvasive adenocarcinoma
Moore SA et al., 2019 [54]	USA	76	Male	Mammary Paget’s disease with underlying ductal carcinoma in situ (DCIS)	Initially diagnosed as Bowen’s disease at outside facility → underwent wide local excision → histopathology revealed Paget’s disease with underlying DCIS
Cen P et al., 2019 [55]	USA	57	Female	Stage IV metastatic ampullary adenocarcinoma	Progression after FOLFOX/FOLFIRI → good response to nab-paclitaxel+gemcitabine±cisplatin
Cen P et al., 2019 [55]	USA	60	Male	Stage IV metastatic ampullary adenocarcinoma	Progression after FOLFOX→FOLFIRINOX+RT → response to nab-paclitaxel+gemcitabine±cisplatin
Cen P et al., 2019 [55]	USA	52	Female	Stage IV duodenal adenocarcinoma with peritoneal & liver metastases	Progression after Whipple surgery + adjuvant FOLFOX → FOLFIRI → salvage nab-paclitaxel + gemcitabine
Kimura T et al., 2020 [56]	Japan	69	Male	Pancreatic head cancer with mixed acinar cell carcinoma (ACC) and ductal adenocarcinoma (DAC) components	Detected as pancreatic head tumor (5–6 cm), underwent pancreatoduodenectomy (Child’s procedure) with lymph node dissection → pathology revealed predominantly ACC with DAC component → treated with adjuvant S-1 chemotherapy for 1 year → no recurrence/metastasis after 9 years follow-up
Fujino R et al., 2020 [57]	Japan	60	Male	Intraductal papillary neoplasm of the bile duct (IPNB) with invasive carcinoma, pancreatobiliary type	Initial liver segmentectomy → recurrence in right hepatic duct at 9 months → right hepatectomy → lung metastases detected 54 months after first surgery → lung metastasectomy ×2 → adjuvant S-1 chemotherapy; patient alive at 64 months
Mai L et al., 2020 [58]	China	72	Male	Prostate adenocarcinoma (Gleason 4+4, ISUP Grade 4) with bulky pelvic lymph node metastases	Initial androgen deprivation therapy (ADT: bicalutamide + goserelin) → PSA nadir at 13.07 ng/mL after 9 months → rapid PSA rise without distant metastasis → developed non-metastatic CRPC (nmCRPC) → next-generation sequencing showed no significant mutation but predicted good docetaxel tolerance → docetaxel 75 mg/m² q21 days × 6 cycles → radiotherapy (IGRT-VMAT 67.5 Gy to prostate, 60–65 Gy to bulky nodes, 47.5 Gy to whole pelvis) → PSA declined continuously to 0.008 ng/mL over 50 months
Mochizuki K et al., 2020 [59]	Japan	88	Female	Carcinosarcoma of the gallbladder (adenocarcinoma + neuroendocrine carcinoma + undifferentiated carcinoma + chondrosarcoma components)	Percutaneous transhepatic gallbladder drainage unsuccessful → cholecystectomy + choledocholithotomy performed → patient declined adjuvant therapy → alive 10 months postoperatively
Koh HH et al., 2020 [60]	Republic of Korea	35	Female	Invasive micropapillary carcinoma (IMPC) of the uterine cervix	Abnormal Pap smear → punch biopsy → preoperative MRI showed no visible mass → underwent radical hysterectomy with bilateral salpingo-oophorectomy and pelvic lymph node dissection → confirmed IMPC
Shinoya Y et al., 2021 [61]	Japan	68	Male	Primary lung adenocarcinoma (stage IIIB → relapsed stage IV)	Initial response to carboplatin+pemetrexed+RT → relapse with multiple metastases → pembrolizumab (2 cycles) → severe melaena → surgical resection of small intestinal metastasis → developed Trousseau syndrome → death on day 174
Oka Y et al., 2021 [62]	Japan	68	Male	Pancreatic ductal adenocarcinoma (tail of pancreas)	Distal pancreatosplenectomy → adjuvant gemcitabine ×6 months → brain + lung metastases at 8 months → gamma knife for brain mets, Ommaya reservoir for cystic lesion → best supportive care
Xiao M et al., 2021 [63]	China	58	Male	Intraductal papillary neoplasm of the intrahepatic bile ducts (IPNB) with high-grade intraepithelial neoplasia, complicated by chronic disseminated intravascular coagulation (DIC) and thrombosis	Initial imaging showed bile duct dilation and a mucin-producing lesion - Hematologic abnormalities (anemia, thrombocytopenia, hypofibrinogenemia) progressed over 1 month - Thrombosis in IVC, pulmonary artery, and atrium developed (similar to Trousseau syndrome) - Anticoagulation improved hematologic parameters - Right hepatic trisectionectomy and thrombectomy performed successfully - Patient recovered well and remained disease-free at 12 months follow-up
Hu W et al., 2022 [64]	China	72	Female	Periampullary adenocarcinoma (pancreatobiliary subtype)	Progression after FOLFOX → good response to gemcitabine + S-1
Shimohama S et al., 2022 [65]	Japan	70	Male	Micropapillary urothelial carcinoma of the bladder, stage IV with lymph node metastases	Initially diagnosed with advanced bladder cancer → chemotherapy (not specified) → developed recurrent cerebral infarctions due to Trousseau’s syndrome → later bowel obstruction due to peritoneal dissemination → palliative colostomy
Kobayashi G et al., 2022 [66]	Japan	92	Female	Mucinous urothelial carcinoma (UC) with clear cell component of the ureter	Gross hematuria → CT showed 2.2 cm papillary lesion in left ureter → urine cytology showed atypical cells with mucin → laparoscopic radical nephroureterectomy performed → histology confirmed mucinous UC with clear cell component → patient disease-free at 3 months
Kang YN et al., 2022 [67]	South Korea	72	Male	Primary clear cell carcinoma of the pancreas	Post-op radiotherapy → local recurrence at 2 months → FOLFIRINOX → hepatic new lesion treated with RFA
Sun Y et al., 2023 [68]	China	50	Female	Clear cell odontogenic carcinoma (CCOC) of right mandible	Initial segmental mandibulectomy + neck dissection → local recurrence at 10 months → 2nd surgery + adjuvant chemoradiation (Taxel-Platin + 64 Gy RT) → distant pleural & vertebral metastases at 24 months → death at 29 months
Kawasaki T et al., 2023 [69]	Japan	65	Female	Neuroendocrine tumor (NET) of the breast with invasive micropapillary carcinoma features	11-month history of breast mass → imaging revealed large cystic tumor with nodal enlargement → mastectomy performed → histopathology confirmed NET with invasive micropapillary pattern → adjuvant endocrine therapy with anastrozole started → alive and disease-free at 12 months
Lu G et al., 2025 [70]	China	65	Male	Gastroesophageal junction hepatoid adenocarcinoma (GEJ HAC) with liver metastasis	AFP elevation → endoscopy revealed a GEJ mass → imaging confirmed liver metastasis
Chang YY et al., 2025 [71]	China	33	Male	Dome-type adenocarcinoma (GALT carcinoma) of the colon	Detected during surveillance colonoscopy, resected with no recurrence at 1 year

The detailed process of study identification, screening, and inclusion is illustrated in the PRISMA flow diagram (Figure 1).

Included Studies Overview

A total of 61 eligible case reports and case series were included in this review, spanning publications from 1999 to 2025. From these reports, a total of 73 individual patient cases were extracted and analyzed. The number of publications increased in recent years, with a notable rise after 2020, suggesting growing interest in TA-MUC1 as a biomarker and therapeutic target.

Geographically, most reports originated from Japan (n = 45, 73.8%), followed by the United States (n = 9, 14.8%), China (n = 6, 9.8%), Turkey (n = 4, 6.6%), and the Republic of Korea (n = 3, 4.1%). This distribution reflects both regional research trends and early clinical adoption of TA-MUC1 immunohistochemistry, particularly in East Asia.

The 73 patients included 41 males and 32 females, with a median age of 65 years (range: 33-92 years). This balanced sex distribution and wide age range indicate that TA-MUC1-positive malignancies are not restricted to a specific demographic group. The included studies provided detailed individual-level data on patient demographics, tumor types, TA-MUC1 expression patterns, treatment histories, and clinical outcomes, enabling a comprehensive narrative synthesis based on 73 cases (Table 3).

**Table 3:** Demographic data of the included articles Data represent aggregated characteristics of 73 patients extracted from 61 case reports or case series published between 1999 and 2025. Age is reported as median (range). Sex distribution is shown as absolute numbers.

Characteristic	Data
Number of publications	61 case reports/series
Total cases	73 cases
Publication years	1999-2025
Median age (range)	65 years (33-92)
Sex distribution	41 males
	32 females

Cancer Types

The 73 included case reports and case series encompassed a broad spectrum of epithelial malignancies, which were classified into 14 major organ-based categories. Among the included TA-MUC1-positive cases, pancreatic cancer was most frequently reported (n = 14, 19.2%), followed by colorectal cancer (n = 10, 13.7%), biliary tract cancer (n = 9, 12.3%), and gastric cancer (n = 6, 8.2%). Urinary tract cancers-including urothelial carcinomas of the bladder, ureter, and renal pelvis-were described in five cases (6.8%), indicating that TA-MUC1 expression has been reported beyond gastrointestinal malignancies. Other cancers represented in the literature included non-small cell lung cancer (n = 4, 5.5%), mesothelioma (n = 3, 4.1%), ovarian cancer (n = 2, 2.7%), and thymic tumors (n = 2, 2.7%). Less frequently reported entities involved the breast, uterus, liver, esophagus, and skin, each described in one or two cases. A small group of unclassified or rare entities (n = 7, 9.6%) was grouped under “Other.” This reporting distribution reflects the cancer types most commonly documented as TA-MUC1-positive in the published literature, rather than indicating prevalence or comparative frequency within all cancers. Therefore, no prognostic inferences can be drawn without TA-MUC1-negative comparators (Table 4).

**Table 4:** Distribution of Reported TA-MUC1-Positive Cancer Types Percentages denote the proportion of each cancer type among the total tumor-associated MUC1 (TA-MUC1)-positive cases (n = 73) and represent reporting density, not prevalence. “Urinary tract cancer” includes urothelial carcinomas of the bladder (n = 3), ureter (n = 1), and renal pelvis (n = 1). “Other” includes rare or unclassified tumors such as neuroendocrine tumors (e.g., breast NET with micropapillary features, n = 1), sarcomatoid carcinomas (e.g., pancreatic sarcomatoid carcinoma, n = 1), and mixed histologies (e.g., combined acinar cell carcinoma and ductal adenocarcinoma of the pancreas, carcinosarcoma of the gallbladder, hepatoid adenocarcinoma of the gastroesophageal junction, and dome-type adenocarcinoma of the colon). Data are derived exclusively from published TA-MUC1-positive case reports and case series.

Cancer Type	Number of Cases	Reporting Density (%)
Pancreatic cancer	14	20.6
Colorectal cancer	10	14.7
Biliary tract cancer	9	13.2
Gastric cancer	6	8.8
Urinary tract cancer	5	7.4
Lung cancer	4	5.9
Mesothelioma	3	4.4
Ovarian cancer	2	2.9
Thymic tumors	2	2.9
Breast cancer	2	2.9
Uterine cancer	1	1.5
Liver cancer	1	1.5
Esophageal cancer	1	1.5
Skin cancer	1	1.5
Other	7	10.3

TA-MUC1 Expression Patterns

Expression sites:* *TA-MUC1 expression was reported in a variety of anatomical contexts. Among the 73 included cases, the majority evaluated expression in the primary tumor site only, which was the sole site of immunohistochemical staining in 26 cases (35.6%). Expression in metastatic lesions alone was reported in 10 cases (13.7%), often involving lymph nodes, peritoneum, or distant organs such as the brain or bone. A subset of cases (five cases, 6.8%) included direct comparisons of both primary and metastatic sites, enabling limited insight into inter-site consistency. Additionally, three cases (4.1%) evaluated TA-MUC1 expression in rare or atypical sites, such as the pleura, adrenal gland, or central nervous system, expanding our understanding of TA-MUC1 distribution beyond traditional epithelial targets. In the remaining cases, the expression site was either not specified in detail or involved composite samples, such as biopsies obtained from post-surgical residual tissue or cytology specimens. These findings suggest that TA-MUC1 expression is not limited to a specific anatomical site and may be detectable in both primary and metastatic lesions, including sites relevant to systemic dissemination and advanced-stage disease (Table 5).

**Table 5:** TA-MUC1 Expression Patterns Summary Percentages are calculated from the total number of tumor-associated MUC1 (TA-MUC1)–positive cases (n = 73). Intensity categories were normalized according to predefined H-score, percent-positive, or qualitative thresholds. Because reporting formats differed, limited overlap between categories may occur. No prognostic inference was made from intensity data in the absence of TA-MUC1–negative comparators.

Expression Pattern Category	Number of Cases	Percentage (%)
Expression Site		
Primary only	26	35.6
Metastatic only	10	13.7
Both primary & metastatic	5	6.8
Rare / atypical sites	3	4.1
Unspecified / mixed	29	39.7
Expression Intensity (normalized)		
Strong (H-score ≥200 or >75%)	20	27.4
Moderate (H-score 100–199 or 26–75%)	8	11
Weak (H-score ≤99 or ≤25%)	6	8.2
Unspecified or qualitative only	39	53.4
Primary vs Metastatic Comparison		
Localized only (not applicable)	17	23.3
No site-specific information	7	9.6
Comparison not feasible	6	8.2
No difference between sites	2	2.7
Other / Unclear	41	56.2

Expression intensity: The reported intensity of TA-MUC1 expression varied considerably among studies, reflecting differences in antibody clones, staining protocols, and scoring criteria. To facilitate cross-study comparison, intensity categories were normalized using predefined cut-points based on the information available in each report. When H-scores were reported, values of 0-99, 100-199, and 200-300 were mapped to weak, moderate, and strong expression, respectively. When only the percentage of positive tumor cells was provided, ≤25% was considered weak, 26-75% moderate, and >75% strong. For qualitative descriptors, terms such as “faint,” “focal,” or “1+” were categorized as weak; “partial,” “intermediate,” or “2+” as moderate; and “diffuse,” “intense,” or “3+” as strong. After this normalization, strong expression was identified in 20 cases (27.4%), moderate expression in eight (11.0%), and weak expression in six (8.2%). An additional 39 cases (53.4%) were reported only as “positive” without sufficient detail for classification. These results highlight substantial heterogeneity in reporting and underscore the need for standardized immunohistochemical scoring systems for TA-MUC1 evaluation in future research (Table 5).

Comparison between primary and metastatic sites:* *Among the 73 included cases, 17 (23.3%) had localized disease without metastasis and were therefore not applicable for inter-site comparison. Seven cases (9.6%) provided no information regarding site-specific assessment, while six (8.2%) explicitly stated that comparative evaluation was not feasible. Only two cases (2.7%) clearly reported paired analyses demonstrating no observable difference in TA-MUC1 expression between the primary tumor and corresponding metastatic lesions. Notably, no reports described discordant expression, such as positivity in the primary tumor and negativity in the metastasis, or vice versa. Given that formal paired assessments were rarely undertaken, the available evidence suggests apparent concordance in the limited paired data available, rather than establishing systemic stability of TA-MUC1 expression across disease sites. Future studies, including larger, paired primary-metastatic cohorts, will be essential to confirm whether TA-MUC1 expression remains stable during tumor progression and dissemination (Table 5).

Treatment Modalities

A wide range of treatment strategies was employed across the 73 TA-MUC1-positive cases. The most frequently reported modality was surgical resection, performed in 47 cases (64.4%), reflecting that many patients were initially diagnosed at a resectable stage or underwent surgery for palliative debulking. Chemotherapy was administered in 41 cases (56.2%), with commonly used regimens including: FOLFOX (5-fluorouracil, leucovorin, and oxaliplatin), gemcitabine/nab-paclitaxel, platinum-based doublets such as cisplatin or carboplatin plus etoposide or pemetrexed, and S-1-based regimens, particularly in Japanese reports. In 14 cases (19.2%), immune checkpoint inhibitors such as nivolumab or pembrolizumab were administered, often in the context of advanced or refractory disease. Of these, a subset co-expressed PD-L1 or had high TMB, although data were limited. Radiotherapy was used in 13 cases (17.8%), either as adjuvant treatment after resection or for palliation of local symptoms such as bone pain or neurologic compression.

Targeted approaches were described in seven TA-MUC1-positive cases (9.6%). Two mechanistically distinct groups were identified. TA-MUC1-directed antibodies and ADCs such as gatipotuzumab (PankoMab-GEX) - a humanized IgG1 antibody recognizing the tumor-associated MUC1 epitope (TA-MUC1). It has been evaluated in phase I/II studies for ovarian and breast cancer (e.g., NCT01222624, NCT01430240) and as an ADC conjugated to cytotoxins in preclinical work. DS-3939a, a TA-MUC1-targeted ADC investigated in early-phase trials (phase I; NCT04189349), was reported in several case studies with variable outcomes. MUC1-C inhibitors such as GO-203, a peptide inhibitor disrupting MUC1-C dimerization, has entered phase I testing (NCT02204085) in advanced solid tumors. None of the included case reports described combination use with chemotherapy or immunotherapy. No evidence from these case reports supports blood-brain-barrier penetration or central nervous system (CNS) efficacy, and conclusions were limited to observed systemic responses [72].

Multimodal treatment approaches were common. Twenty-one cases (28.8%) received both surgery and chemotherapy. Twelve cases (16.4%) received trimodal therapy involving surgery, chemotherapy, and radiotherapy or immunotherapy. Seven cases (9.6%) received experimental therapies in addition to standard treatment (Table 6).

**Table 6:** Treatment Modalities in TA-MUC1-Positive Cases Percentages were calculated based on the total number of included cases (n = 73). Some patients received more than one treatment modality. “Trimodal” refers to the combination of surgery, chemotherapy, and either radiotherapy or immunotherapy. “Standard + Experimental” includes patients who received investigational therapies such as tumor-associated MUC1 (TA-MUC1)-targeted ADCs in addition to conventional treatment approaches. ADC = antibody-drug conjugates, IO/RT = immunotherapy/radiation therapy

Treatment Modality	Number of Cases	Percentage (%)
Surgery	47	64.4
Chemotherapy	41	56.2
Immunotherapy	14	19.2
Radiotherapy	13	17.8
Targeted therapy (including ADCs)	7	9.6
Surgery + Chemotherapy	21	28.8
Trimodal (Surgery + Chemo + IO/RT)	12	16.4
Standard + Experimental	7	9.6

Survival outcomes:* *Survival outcomes were heterogeneously reported across the 73 TA-MUC1-positive cases. When available, progression-free survival (PFS) and overall survival (OS) were expressed in months. Cases without progression or death at last follow-up were treated as censored, and their follow-up duration was recorded as the observed PFS or OS, respectively. All estimates below are strictly descriptive; no pooling or inferential statistics were performed.

Progression-free survival:* *PFS was available in 56/73 (76.7%) cases. Among these, 47/56 (83.9%) had a documented progression event, whereas 9/56 (16.1%) were censored (progression-free at last follow-up). The median PFS was 12.0 months (IQR 6.0-19.5; range 0.0-118.0 months).

Overall survival: OS was available in 56/73 (76.7%) cases. Among these, 14/56 (25.0%) deaths were reported and 42/56 (75.0%) observations were censored (alive at last follow-up). The median OS was 12.0 months (IQR 8.0-26.0; range 0.5-118.0 months).

Co-existing Biomarkers

Data on co-existing molecular biomarkers were available for a limited subset of the 73 TA-MUC1-positive cases and are summarized descriptively. Biomarker analyses were generally performed to inform treatment selection (immunotherapy or targeted therapy). An evidence map (Table 7) presents the frequency and positivity of each reported biomarker.

**Table 7:** Co-existing Biomarkers in TA-MUC1-Positive Cases Data are derived from a subset of the 73 tumor-associated MUC1 (TA-MUC1)–positive cases for which co-existing biomarker results were explicitly reported. Each biomarker tile is represented by the number of cases assessed (Cases Reported) and the number or proportion of positive findings (Positive Cases, Positive Rate). Findings are strictly descriptive and intended to illustrate the limited evidence base for co-expression patterns between TA-MUC1 and other biomarkers. Mechanistic interpretations (e.g., immune evasion or predictive interactions) are hypothesis-level only and not inferential. Abbreviations: MSI = microsatellite instability; MSS = microsatellite stable; TMB = tumor mutational burden; ICI = immune checkpoint inhibitor; NSCLC = non–small cell lung cancer. Data are derived from individual case reports and case series included in this systematic review, such as Yu J et al. (2010) [29], Cen P et al. (2019) [55], Kimura T et al. (2020) [56], Fujino R et al. (2020) [57], and others (see References [10-71]).

Biomarker	Cases Reported (n)	Positive Cases (n)	Positive Rate (%)	Cancer Types (Examples)	Notes
PD-L1	9	5	55.6	NSCLC, biliary tract	Co-expression with TA-MUC1; ICIs used (variable response)
Microsatellite Instability (MSI)	5	1	20	Colorectal	1 MSI-high case responded to ICI; others MSS
Tumor Mutational Burden (TMB)	3	0	0	Gastrointestinal	All TMB-low; descriptive only
HER2	4	4	100	Gastric, breast	HER2-targeted therapy used; co-expression observed

PD-L1 expression:* *PD-L1 status was reported in nine cases, of which five were positive. Co-expression with TA-MUC1 occurred mainly in non-small-cell lung and biliary tract cancers. Immune checkpoint inhibitors (nivolumab or pembrolizumab) were used in several PD-L1-positive cases, with variable clinical outcomes. While co-expression may suggest a potential immune-evasive phenotype, this should be regarded as a hypothesis-level observation given the limited data.

Microsatellite instability:* *MSI was described in five cases (one MSI-high, four MSS). The MSI-high case, a colorectal carcinoma, showed a favorable response to ICI therapy; no pattern can be inferred from the remaining cases.

Tumor mutational burden:* *TMB was reported in three cases, all of which were TMB-low gastrointestinal tumors. These descriptive findings suggest that TA-MUC1 expression can occur across tumors with differing mutational burdens, but no predictive relationship is implied.

HER2 expression:* *HER2 overexpression was documented in four cases (gastric = two, breast = one, other = one). HER2-targeted therapy (trastuzumab) was combined with chemotherapy in these cases. The coexistence of TA-MUC1 and HER2 was descriptively observed but cannot yet be interpreted as biologically or therapeutically synergistic.

Rare Sites and Case Highlights

In addition to conventional epithelial tumor locations, several cases in this review documented TA-MUC1 expression in unusual or rare anatomical sites, supporting the notion that TA-MUC1 may be expressed beyond classic primary tumors and has relevance in advanced or metastatic disease.

Central nervous system involvement:* *TA-MUC1 positivity was detected in two cases of brain metastases, originating from breast and lung primaries, respectively. In both instances, TA-MUC1 was detected via immunohistochemistry on metastatic CNS tissue. These findings suggest that TA-MUC1-targeted agents may cross the blood-brain barrier or be relevant for tumors with high CNS tropism.

Skeletal metastases:* *Bone involvement, including the vertebrae, femur, and pelvis, was reported in three cases. In all instances, TA-MUC1 staining was retained in the metastatic bone lesions. This is of particular interest given the frequent skeletal metastasis observed in breast, prostate, and lung cancer, and underscores the need to evaluate TA-MUC1 in these contexts.

Pleural and peritoneal dissemination: TA-MUC1 expression was observed in cases of pleural mesothelioma (epithelioid subtype, n = three), as well as in peritoneal metastases from gastrointestinal primaries. In mesothelioma, TA-MUC1 positivity challenges the notion that this marker is restricted to glandular epithelium, suggesting it may have diagnostic utility.

Adrenal gland metastasis:* *One case described strong TA-MUC1 expression in an adrenal metastasis from a colorectal primary. This case demonstrated concordant staining between the primary tumor and the adrenal lesion, reinforcing the marker’s consistency across metastatic sites.

Multifocal cystic and mixed morphologies: Several cases, especially those involving the hepatobiliary and pancreatic systems, described TA-MUC1 expression in multifocal cystic lesions, including intraductal papillary mucinous neoplasms (IPMNs) and hepatic cystadenocarcinomas. This demonstrates that TA-MUC1 can be retained in non-solid and partially differentiated lesions.

Histologically unusual tumors: TA-MUC1 positivity was noted in tumors with sarcomatoid, signet-ring, or poorly differentiated histology, suggesting its utility even in cases lacking classical glandular structure. In particular, sarcomatoid carcinomas of the pancreas and lung retained strong TA-MUC1 staining despite dedifferentiation (Table 8).

**Table 8:** Rare Sites and Case Highlights Tumor-associated MUC1 (TA-MUC1) expression was confirmed by immunohistochemistry (IHC) in all cases listed. “Primary Tumor Type” refers to the original cancer site from which the metastasis or dissemination occurred. Cases with “Cystic/Mixed Morphology” include intraductal papillary mucinous neoplasms (IPMNs) and mucinous cystadenocarcinomas. “Histologically Unusual Tumors” include non-classical glandular morphologies such as sarcomatoid or signet-ring cell carcinomas. This table highlights the anatomical and histological diversity of TA-MUC1-positive lesions and underscores the potential utility of TA-MUC1-targeted therapies in both typical and atypical presentations. Data are derived from the included case reports and case series, such as Higashi M et al. (1999) [10]; Yamamoto M et al. (2002) [13]; Terada T et al. (2003) [14]; Sakamoto K et al. (2005) [16]; Mogi A et al. (2009) [27]; Kusafuka K et al. (2010) [28]; Yu J et al. (2010) [29]; Ohe M et al. (2011) [30]; Mimatsu K et al. (2011) [31]; Doi H et al. (2011) [32]; Terada T et al. (2012) [33,35,36]; Zen Y et al.(2012) [34]; and others (see References [10-71]).

Site / Feature	Number of Cases	Primary Origin (Examples)	Notes
Central Nervous System (CNS)	2	Breast, Lung	TA-MUC1 IHC positive in brain metastases
Skeletal Metastases	3	Lung, Breast, Colorectal	Retained TA-MUC1 in bone lesions
Pleural Mesothelioma	3	Epithelioid Mesothelioma	TA-MUC1 positive in epithelioid type
Peritoneal Metastases	4	Gastric, Colorectal	Observed in peritoneal metastasis tissue
Adrenal Gland Metastasis	1	Colorectal	Concordant with primary colorectal tumor
Cystic/Mixed Morphology	5	Pancreatic, Hepatobiliary	Includes IPMNs, hepatic cystadenocarcinoma
Histologically Unusual Tumors	6	Pancreas, Lung, Unknown	Includes sarcomatoid and signet-ring types

Quality Assessment Results

All 61 included publications, comprising 73 individual cases, were assessed for methodological quality using the JBI Critical Appraisal Checklist for Case Reports and Case Series. The following domains were systematically evaluated: Clear description of patient demographics and clinical history, Accurate diagnosis and confirmation methods, Detailed reporting of treatment modalities, Outcomes and follow-up duration, and Documentation of histopathological/immunohistochemical findings, including TA-MUC1 expression in Case Reports (n = 47 articles).

Among the case reports, most met the majority of JBI criteria. 95.7% (45/47) clearly reported clinical presentation, diagnostic process, and therapeutic interventions. 87.2% (41/47) described TA-MUC1 expression in adequate pathological detail (e.g., site, staining intensity, detection method). However, long-term follow-up was missing or limited in 40.4% (19/47) of reports. Only 29.8% (14/47) discussed potential limitations or addressed confounding factors such as co-existing biomarkers or prior treatments.

Case series (n = 14 articles):* *For case series, quality was generally higher due to structured data presentation across multiple patients. One hundred percent (14/14) provided consistent clinical and pathological descriptions across all included cases. 71.4% (10/14) included at least partial outcome data (e.g., PFS, OS, or recurrence). Some lacked explicit inclusion criteria or methodological transparency in case selection, which may introduce reporting bias.

Overall quality considerations:* *While most studies met fundamental criteria for validity and clinical utility, certain limitations were common: heterogeneous reporting formats, especially in older reports; incomplete survival data, particularly OS or PFS estimates; and sparse reporting of co-biomarker status (e.g., PD-L1, MSI, TMB).

Despite these limitations, the overall quality of included studies was deemed moderate to high, and sufficient to support narrative synthesis of TA-MUC1 expression, associated treatments, and clinical outcomes.

Discussion

Summary of the Study

This systematic review analyzed 73 individual cases of TA-MUC1-positive malignancies reported across 61 publications between 1999 and 2025. By extracting detailed clinical and pathological data from case reports and series, we revealed the real-world diversity of tumors that express TA-MUC1, ranging from common gastrointestinal cancers to rare tumors such as sarcomatoid carcinomas, mesotheliomas, and adrenal metastases.

Importantly, TA-MUC1 expression was documented not only in primary lesions but also in metastatic and poorly differentiated tumors, including those involving the brain, bone, and adrenal glands. The staining pattern was often preserved across disease stages, and in some cases, increased intensity of TA-MUC1 expression was associated with disease progression. This consistency suggests that TA-MUC1 may serve as a stable molecular marker throughout the disease course.

The analysis also shed light on treatment trends, as many patients underwent multimodal therapies, including surgery, systemic chemotherapy, and targeted agents. A subset received immunotherapy, and some were treated with experimental regimens. While the median progression-free and overall survival times were modest, long-term control spanning several years was documented in multiple cases, especially in those with complete resection or durable responses to systemic treatment.

Comparison With Other Studies

TA-MUC1 has been widely studied as a tumor-associated antigen, particularly in breast and pancreatic cancer, where it is known to be overexpressed in a hypoglycosylated, immunogenic form [73-75]. Previous large-scale studies and clinical trials have focused on TA-MUC1 as a therapeutic target for vaccines, monoclonal antibodies, and, more recently, ADCs such as DS-3939a and GO-203 [72,76].

However, most of these studies have focused on specific tumor types or patient populations [76,77]. In contrast, our case-based review demonstrates that TA-MUC1 is not organ-specific but rather a broadly expressed molecule in epithelial malignancies. The detection of TA-MUC1 in CNS metastases, cystic biliary tumors, and sarcomatoid carcinomas expands the known landscape of TA-MUC1-expressing tumors. These findings align with recent reports suggesting that tumor-agnostic targeting of antigens, such as TA-MUC1, may be feasible and clinically relevant, particularly in advanced or treatment-refractory cancers [78-80].

Moreover, our observation that TA-MUC1 can co-exist with other biomarkers, such as PD-L1 and HER2, supports emerging strategies that combine ADCs with immune checkpoint blockade or targeted kinase inhibitors, even in histologically heterogeneous settings [81-83].

Strengths of the Study

One of the significant strengths of this study is its comprehensive narrative synthesis of diverse and granular data. Unlike randomized controlled trials, case reports often capture clinically significant but rare phenomena-such as strong biomarker expression in unusual histology or rare metastatic patterns-which are challenging to assess in large datasets. The study demonstrates TA-MUC1 expression across a wide range of histological spectra, including glandular, solid, poorly differentiated, and sarcomatoid morphologies. It identifies stable expression of TA-MUC1 in both primary and metastatic lesions, suggesting it may serve as a reliable therapeutic target throughout the disease course. The dataset reveals possible biomarker co-expression patterns that could inform future combination strategies (e.g., TA-MUC1 + PD-L1 inhibitors). It provides real-world evidence of treatment outcomes, highlighting potential clinical benefits of surgery and multimodal therapy in selected cases.

Limitations

This study has several significant limitations. First, the exclusive reliance on case reports and case series introduces selection and publication bias; unusual or positive findings are more likely to be reported. Second, there was marked heterogeneity in TA-MUC1 immunohistochemical assessment, including variability in antibody clones, staining protocols, and scoring methods, which precludes meaningful comparison of expression intensity across cases. Third, the total number of reported patients (n = 73) is small and geographically skewed, with most reports originating from Japan, which limits generalizability. Fourth, survival outcomes (PFS, OS) were inconsistently reported and highly susceptible to bias; these should be regarded as illustrative only. Finally, the lack of TA-MUC1-negative controls prevents evaluation of independent prognostic or predictive value. These limitations emphasize that the present findings are exploratory and hypothesis-generating rather than definitive.

## Conclusions

This review indicates that TA-MUC1 expression has been reported across a broad spectrum of epithelial malignancies, including rare and metastatic sites. That expression is often preserved in advanced or dedifferentiated states. While these descriptive findings suggest that TA-MUC1 could serve as a potential therapeutic target, the current evidence is limited by small sample sizes, heterogeneity of methods, and reporting bias. Therefore, the results should be interpreted with caution and considered hypothesis-generating. Prospective, large-scale studies with standardized TA-MUC1 evaluation and integrated biomarker analyses are essential to establish its clinical relevance in precision oncology and the development of ADCs.
